# 

**Published:** 1885-06

**Authors:** 


### BOOKS RECEIVED.

*Modern Therapeutics of the Diseases of Children*, by Joseph F. Edwards, M. D. Philadelphia: D. G. Brinton.

*Minor Surgical Gynecology*, by Paul F. Mundé, M. D. New York: William Wood & Co. Chicago: W. T. Keener.

*A Practical Treatise on Nasal Catarrh and Allied Diseases*, by Beverly Robinson, A. M., M. D. (Paris). Second Edition. New York: William Wood & Co. Chicago: W. T. Keener.

*Handbook of Diseases of the Skin*, edited by H. von Ziemssen. New York: William Wood & Co. Chicago: W. T. Keener.

*The Oleates: An Investigation into Their Nature and Action*, by John V. Shoemaker, A. M., M. D. Philadelphia: H. A. Davis, Attorney.

*Injuries of the Spine and Spinal Cord, without Apparent Mechanical Lesion and Nervous Shock, in their Surgical and Medico-Legal Aspects*, by Herbert W. Page, M. A., M. C. (Cantab.) Second Edition. Philadelphia: P. Blakiston, Son & Co.

*Transactions of the New York State Medical Association*, for the year 1884. Vol. I.

*The Curability and Treatment of Pulmonary Phthisis*, by S. Joccond. Translated by Montague Lubbock. New York: D. Appleton & Co. Chicago: Jansen, McClurg & Co.

*Handbook of Physiology*, by W. Morrant Baker, F. R. C. S., and Vincent Dormer Harris, M. D. Vol. II. New York: William Wood & Co. Chicago: W. T. Keener.

*Clinical Studies on Diseases of the Eye*, by Dr. Ferdinand Ritter von Arlt. Translated by Lyman Ware, M. D. Philadelphia: P. Blakiston, Son & Co. Chicago: W. T. Keener.

### PAMPHLETS RECEIVED.

---

Medizinische Jahrbücher herausgegeben von der K. K. Geselschaft der Aerzte Redigirt von Professor E. Albert, Professor H. Kundrat und Professor E. Ludwig. Jahrgang, 1884, IV Heft.

Disinfection and Disinfectants. Preliminary Report made by the Committee on Disinfectants of the American Public Health Association.

Dr. Seguins' Metric Prescription Book. G. P. Putnam's Sons.

Insanity and Divorce. The Neuropathic Condition and Treatment of Cancer. Mysomania, by C. H. Hughes, M. D. Abstracted Editorials from the *Alienist and Neurologist.*

A Case of Psycho-Sensory (affective or moral) Insanity, by C. H. Hughes, M. D. Reprint, *Alienist and Neurologist.*

A Lecture on Sterility, by William H. Walthen, M. D.

565

### INDEX TO VOLUME L.

### A.

PAGE.

Abortion, Missed, O'Connell ... 321
Abscess, Peri-Uterine, Fenger . 504
Absorption of Salicylic Acid, Editorial ... 237
Abstracts and Extracts ... 460
A case of Mural Pregnancy, Byford ... 1
Actinimycosis in the Human Subject, Murphy ... 259
Albuminuria, Picric Acid Test for, Cary ... 9
A Manual of Diseases of the Throat and Nose, Rinew ... 247
Amenorrhoea, Permanganate of Potassium in, Doering 283, 449
American Medical Association . 377
American Medical Association, Editorial ... 440
An Easy Method of External Pelvic Measurement, Editorial ... 301
Apomorphia, Muriate of, Webster ... 323
A Simple Microtome, Webster . 12
Asthma, Muriate of apomorphia in ... 323
Atlee Washington L., Editorial 235

### B.

Babcock R. H., Review by ... 20
Bettman Boerne, Peroxide of Hydrogen ... 318
Translation ... 91
Translation ... 485
Books Received ... 280, 373, 562
Book Reviews . 20, 144, 247, 370, 452
Botany, Medical, Review ... 371
Braithwaite, William, Death of 246
Breech Presentation ... 293

PAGE

Brights Disease, Pre-Albuminuric stage of, Purdy ... 379
Byford, Henry T., Membranes in Labor ... 226
Labor ... 276
Byford, William H., Mural Pregnancy ... 1

### C.

Caldwell, Charles, Two Interesting Cases in Obstetrics ... 291
Cary, Frank, Picric Acid test for Albuminuria ... 9
Casselberry, W. E., Review ... 251
Chaffee, C. W., In Memoriam . 267
Chancre of the Eyelid, Holmes 296
Chapman G. H., Diphtheria 128, 268
Chicago Gynecological Society Meetings :
Dec. 19th, 1884. ... 58
Jan. 16th, 1885 ... 177
Feb. 20th, 1885 ... 275
Mar. 20th, 1885 ... 444
April 19th, 1885 ... 504
Chicago Medical Society Meetings :
Dec. 1, 1884 ... 67
" 15, 1884 ... 259
Jan. 5, 1885 ... 267
" 19, " ... 312
Feb. 2, " ... 318
" 16, " ... 324
Mar. 2, " ... 327
April 6, " ... 377
Chicago Medical College, Commencement Exercises ... 474
Children, Diseases of, Review . 20
Chloroform, External use of, in Labor, Editorial ... 14
Cimicifuga Racemosa, Knox ... 287

PAGE

Clevenger S. V., Item ........ 19
Clinical Reports.............. 365
Cocaine as an Anæsthetic, Koller ........................ 91
Colleges of Physicians and Surgeons, Commencement of... 247
College of Physicians of Philadelphia :
Meeting May 6, 1885........ 534
Comma Bacillus, McArthur... 327
Correspondence
24. 146, 254, 307, 438
Cramp of the Levator Ani.... 467
Croup, Treatment of.......... 475

D.

Davis, C. G., Tape worm in a child ........................ 325
Delafield, Francis, Review.... 558
Diphtheria................... 268
Diphtheria, Chapman.......... 128
Diseases of the Nose, Review.. 556
Doering, Edmund J., Permanganate of Potassium in Amenorrha ...................283, 449
Dr. Warren's Advice.......... 438
Dudley, C., Double Ovariotomy, 177
Dudley, E. C., Leio-myoma.... 276

E.

Eczema, Treatment of, Reynolds ........................ 492
Editorials..14, 139, 235, 300, 440, 500
Emmenagogue, Manganese as an, Martin.................. 119
Errata ........................ 143
Extensive injury of the Face, Tilley ...................... 75
Extra Uterine Pregnancy, Fenger ......................... 211
Extra Uterine Pregnancy, Jaggard ....................... 78
Extra Uterine Pregnancy, Operation for, Editorial.......... 501

F.

Fenger, Christian, Report..... 211
Fenger, Christian, Peri-Uterine Abscess....................... 504
Functions of the Membranes in Labor, Byford............226, 276

G.

PAGE

Gradle, Henry, Neuroses, etc.. 430
Greene, J. Henry, Review..... 554
Groesbeck, Abram, Resolutions, 67
Gynæcological Teaching on the Phantom.................... 468

H.

Hard, Abner, Death of.........378
Hearing—Observations on determining the Power of..... 469
Heuck's Atlas of Surgical Anatomy, Review................. 252
Histology, Manual of, Review. 561
Hoadly, A. E., New Method of Counting a Rapid Pulse..... 315
Holden's, Anatomy, Review.... 144
Holmes, E. L., Chancre of the Eyelid...................... 296
Homans, John, Letter......... 257
Home for Incurables in Chicago, 243
Hooper's Physicians Vade Mecum ..................... 373
Hyde, James Nevins, Skin Affections.................... 187
Hyde, James Nevins, Resignation ..................... 235
Hydrargyrum Tannicum...... 113
Hygiene, Text-book of, Review, 456
Hygroma of the Tongue, Zeisler, 326

I.

Illinois Physicians............ 17
Illinois State Medical Society. 246
Review of Transactions of,.. 452
34th Annual Meeting....... 513
Editorial.................. 500
Illinois State Board of Health
.......................46, 244
Index Medicus............... 245
Insane Hospitals, Fires in, Editorial ..................... 239
International Medical Congress, 42
Intestinal Obstruction......... 315
Intestinal Obstruction, Review. 559
Intra-Mural Leio-myoma, Dudley ...................... 276
Irresponsible Opinion, Editorial...................... 300
Is Piric Acid a Reliable and Practical test for Albuminuria, Cary.................. 9

### J.

PAGE
Jaggard, W. W., Extra Uterine Pregnancy ... 78
Jaggard, W. W., Teratom ... 511
Johnson, Lawrence, Review ... 371

### K.

Koller, Charles, Cocaine ... 91
Knox, J. Suydam, Cimicifuga Racemosa in Parturition ... 287

### L.

Labia Minora, Anatomy and Pathology of ... 456
Labor and Child-Bed in Elderly Primiparæ ... 460
Labor, External use of Chloroform in, Editorial ... 14
Labor, Functions of the Membranes in, Byford ... 226, 276
Lagorio, A., Letter ... 37, 146, 307
Lagorio, A., Fibrinous Pneumonia ... 369
Lagorio, A., Translations ... 163
Laparo-Myotomy ... 464
Lectures on the Principles of Surgery, Review ... 144
Leed's, Dr., Criticism of paper by Meigs ... 534
"Let not the Cobbler go Beyond His Last," Editorial ... 139
Letter from Edinburgh ... 151
Letter from Italy ... 37
Letter from Italy ... 146
Letter from Wiesbaden ... 30
Literary Notices ... 142
Little, James Lawrence, Death of ... 378
Local News ... 176, 243, 377, 474, 564

### M.

Mackenzie, Morell, Review ... 247
Maisch, John M., Review ... 555
Manganese as an Emmenagogue, Martin ... 119
Martin, Franklin H., Manganese as an Emmengogue ... 119
Materia Medica, Organic, Review ... 555
McArthur, L. L., Comma Bacillus ... 327
Medical Colleges, Gifts to ... 41
Membranes in Labor, Byford ... 226
Mercury, Tannate of, Zeisler ... 312
PAGE
Microtome, A simple, Webster ... 12
Mollities Ossium, Webb ... 327, 414
Murphy, J. B., Actinimycosis ... 259

### N.

Nervous Symptoms due to Overlooked Anomalies of the Eyes and Ears, Gradle ... 430
New Remedies, Editorial ... 140
News Items ... 17, 245

### O.

Objective Diagnosis, Editorial ... 442
Obstetrics and Gynecology, Extracts ... 460
O'Connell, P., Missed Abortion, 321
Ohio State Sanitary Association, 19
On the Affections of the Skin Induced by Temperature Variations in Cold Weather, Hyde ... 187
Original Communications ... 1, 101, 187, 283, 378, 475
O'Shea, David, Typhoid Fever, 330
Ovariotomy, Interesting Double, Dudley ... 177
Ovariotomy, Report of Three Operations, Steele ... 101
Ovariotomy, Revival of, Editorial ... 235
Ovariotomy, Spray in, Homans 257

### P.

Pamphlets Received ... 280, 375, 563
Parturition, Cimicifuga Racemosa in, Knox ... 287
Pathological Anatomy and Histology, Review ... 558
Pathological Anatomy, Text Book of, Review ... 552
Pathologists' Fees, Editorial ... 237
Pathology and Morbid Anatomy, Review ... 554
Pathology, Practical, Review ... 553
Permanganate of Potassium, Doering ... 283, 449
Peroxide of Hydrogen, Bettman ... 318, 485
Personals ... 473, 378
Phosphides and Sulphides, Edtorial ... 142
Physicians' Protective Association, Editorial ... 15
Physiological Physics, Review ... 370
Picric Acid as a test for Albuminuria, Cary ... 9

PAGE
Placenta, Accidental Separation of ........ 291
Pneumonia, Fibrinous ........ 395
Pre-Albuminuric Stage of Chronic Bright's Disease ........ 379
Pregnancy, Mural, Byford ........ 1
Presbyterian Hospital ........ 564
Psoriasis, a clinical lecture, Reynolds ........ 7
Pulse Rapid, New Method of Counting, Hoadley ........ 315
Purdy, Chas. W., Letters, 24, 151, 254
Purdy, Chas. W., Bright's Disease ........ 378

R.

Remote Puerperal Hæmorrhage ........ 431
Reynolds, Henry J., Psoriasis ........ 7
Reynolds, Henry J., Eczema ........ 492
Robertson, J. McGregor, Review ........ 370
Rohe, Geo. H., Hygiene, Review ........ 456
Rush Medical College Commencement ........ 176

S.

Salicylic Acid, Absorption of, Editorial ........ 237
Sanitary Council of the Mississippi Valley ........ 246
Sanitary Inspection, Editorial ........ 304
Selections ........ 40, 174
S. H. S., Letter ........ 30
Sims, J. Marion, Review of Autobiography ........ 453
Skin Diseases. Hand-book of, Review ........ 455
Smith, Eustace, Review ........ 20
Smith, Wm. M., Translation ........ 157
Society Proceedings ........ 58, 177, 312, 444, 504
Some Remarks on the Value of Permanganate of Potassium in Amenorrhoea, Doering ........ 283
Steele, D. A. K., Ovariotomy ........ 101
St. Luke's Hospital ........ 245
Suicide, Editorial ........ 305

T.

Tape Worm in a child, Davis ........ 325
Teratom, presentation of a specimen of, Jaggard ........ 511

PAGE
The Bacilli of Syphilis and Hydrargyrum Tannicum Oxydulatum, Zeisler ........ 113
The Influence of Cimicifuga Racemosa on Parturition, Knox ........ 287
The Pennsylvania Anatomical Act, Selection ........ 174
The Story of my Life, Review 453
Thyroid Gland, Malignant Disease of, Webster ........ 324
Tilley, Robert, Extensive inquiry of the face ........ 75
Tissue-Change in the Fœtus ........ 468
Tubercle Bacilli, Webb ........ 334
Translations ........ 91, 157
Treatment of Croup ........ 475
Treatment of Eczema, Reynolds ........ 492
Treres, Frederick, Review ........ 559
Tuberculosis, Contagiousness of, Webb ........ 334
Two Interesting cases in Obstetrics, Caldwell ........ 291
Typhoid Fever, O'Shea ........ 330

U.

University of Aberdeen, Letter 24
Uterine Hæmorrhage, Removal of clots in ........ 466

V.

Van Buren, W. H., Review ........ 144
Van Harlingen, Arthur, Skin Diseases, Review ........ 455

W.

Wagner, Clinton, Review ........ 556
Waxham, F. E., Croup ........ 475
Webb, W. J., Mollities Ossium ........ 327, 414
Webb, W. H., Tubercle Bacilli 334
Webster, C. E., A Simple Microtome ........ 12
Webster, C. E., Malignant disease of the Thyroid Gland ........ 324
Webster, G. W., Muriate of Apomorphia ........ 323
Wilson, Sir Erasmus, Will of ........ 40
Woodhead, G. Sims, Review ........ 553

Z.

Zeigler, Ernst, Review ........ 552
Zeisler, Joseph, Bacilli of Syphilis ........ 113, 312
Hygroma of the Tongue ........ 326

SUBSCRIPTION PRICE, $3.00 PER YEAR.

Whole Number, 306. JUNE, 1885. Vol. L., Number 6.

LIBRARY
INDEXED
S.G.O.

### THE CHICAGO MEDICAL JOURNAL AND EXAMINER

EDITORS:

S. J. JONES, M.D., LL.D.

W. W. JAGGARD, A.M., M.D.

HAROLD N. MOYER, M.D.

COLLABORATORS.

CHICAGO:

PROFESSOR J. A. ALLEN, M. D., LL. D.
PROFESSOR E. ANDREWS, M. D., LL. D.
PROFESSOR W. H. BYFORD, A. M., M. D.
PROFESSOR O. C. DEWOLF, A. M., M. D.
PROFESSOR E. C. DUDLEY, A. B., M. D.
PROFESSOR J. H. ETHERIDGE, A. M., M. D.
PROFESSOR C. FENGER, M. D.
PROFESSOR J. N. HYDE, A. M., M. D.
PROFESSOR H. A. JOHNSON, M. D., LL. D.
CHARLES GILMAN SMITH, A. M., M.D
J. F. TODD, M. D.

MILWAUKEE, WISCONSIN:
N. SENN, M, D.

WASHINGTON, D. C:
S. O. RICHEY, M. D.

UNITED STATES NAVY:
MEDICAL DIRECTOR J. M. BROWNE, M. D.

CHICAGO:

